# 598. Area under the curve-guided vancomycin monitoring and risk of nephrotoxicity in non-Staphylococcus aureus infections: A case-control study

**DOI:** 10.1093/ofid/ofac492.650

**Published:** 2022-12-15

**Authors:** Douglas Mazewski, Christie M Bertram, Nathaniel J Rhodes, W Justin Moore

**Affiliations:** Northwestern Medicine, Chicago, Illinois; Northwestern Memorial Hospital/Rosalind Franklin University of Medicine and Science, Chicago, Illinois; Midwestern University, Downers Grove, Illinois; Northwestern Medicine, Chicago, Illinois

## Abstract

**Background:**

Area under the curve (AUC)-guided monitoring of vancomycin for the treatment of *Staphylococcus aureus* (SA) infections demonstrates reduced rates of nephrotoxicity in clinical studies. Many institutions utilize AUC-guided dosing for all patients receiving vancomycin, despite extrapolated AUC goals for non-SA infections. We sought to define risk factors for vancomycin-induced kidney injury (VIKI) in non-SA infections.

**Methods:**

This was a retrospective, single-center, case-control study evaluating risk factors associated with VIKI in hospitalized patients receiving AUC-guided dosing for non-SA infections from 2/2019-10/2021. Cases were defined as patients with non-SA infections who received >72 hours of vancomycin and had >1 observed vancomycin concentration with a corresponding AUC calculated who developed VIKI within 24 hours of vancomycin discontinuation. Controls had to meet all case criteria but did not have VIKI. Univariate and multivariable analyses were used to identify risk factors associated with VIKI. Multivariable analyses were conducted using the Optimal Data Analysis package for R.

**Results:**

Twenty-three cases and 48 controls were included. Leading indications were bloodstream (52.1%) and severe skin/skin structure (46.5%) infections. Isolated pathogens from culture included coagulase-negative S*taphylococcus* (49.3%) and *Streptococcus* spp. (43.7%). The majority of vancomycin AUCs (52%) were collected within 24-48 hours of initiation. The median first and second AUCs were higher in cases vs. controls [429 vs. 385 mg*hr/L, p=0.013; and 573 vs. 429 mg*hr/L, p=0.021], respectively. Most patients (75%) experienced AKI within 72 hours of vancomycin initiation, with a median time to AKI of 5 days (IQR 2.5-7). Risk factors associated with AKI included any AUC exceeding 515 mg*hr/L, infection caused by pathogens other than *Streptococcus* spp., and non-bacteremia indications. Patients with AUCs exceeding 515 mg*hr/L had 3.7-fold greater odds of VIKI (p=0.017).

**Conclusion:**

We found that higher AUCs increased the risk of VIKI in non-SA infections and that the risk of VIKI varied by vancomycin indication. Our findings agree with previous studies in SA indicating that vancomycin AUC should not generally exceed 515 mg*hr/L.

**Disclosures:**

**Nathaniel J. Rhodes, PharmD MS**, Paratek: Grant/Research Support|Third Pole Therapeutics: Advisor/Consultant.

